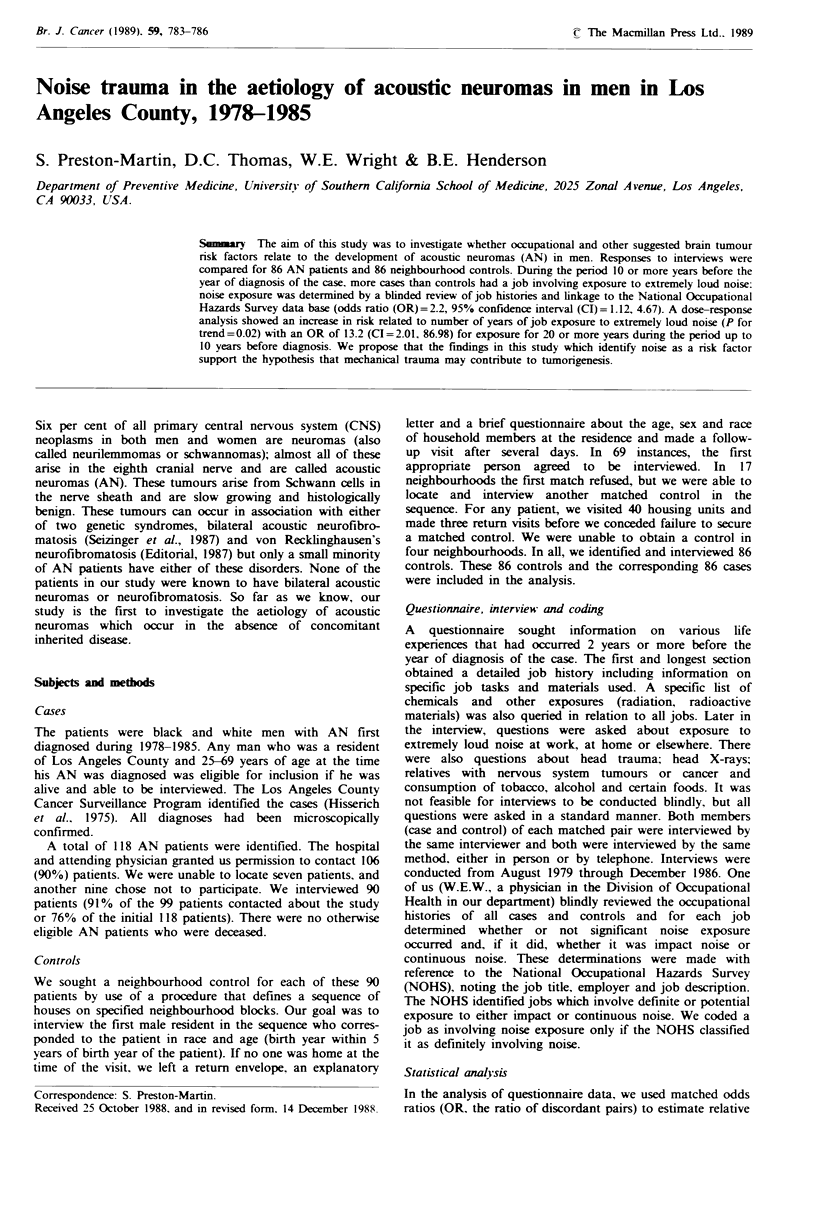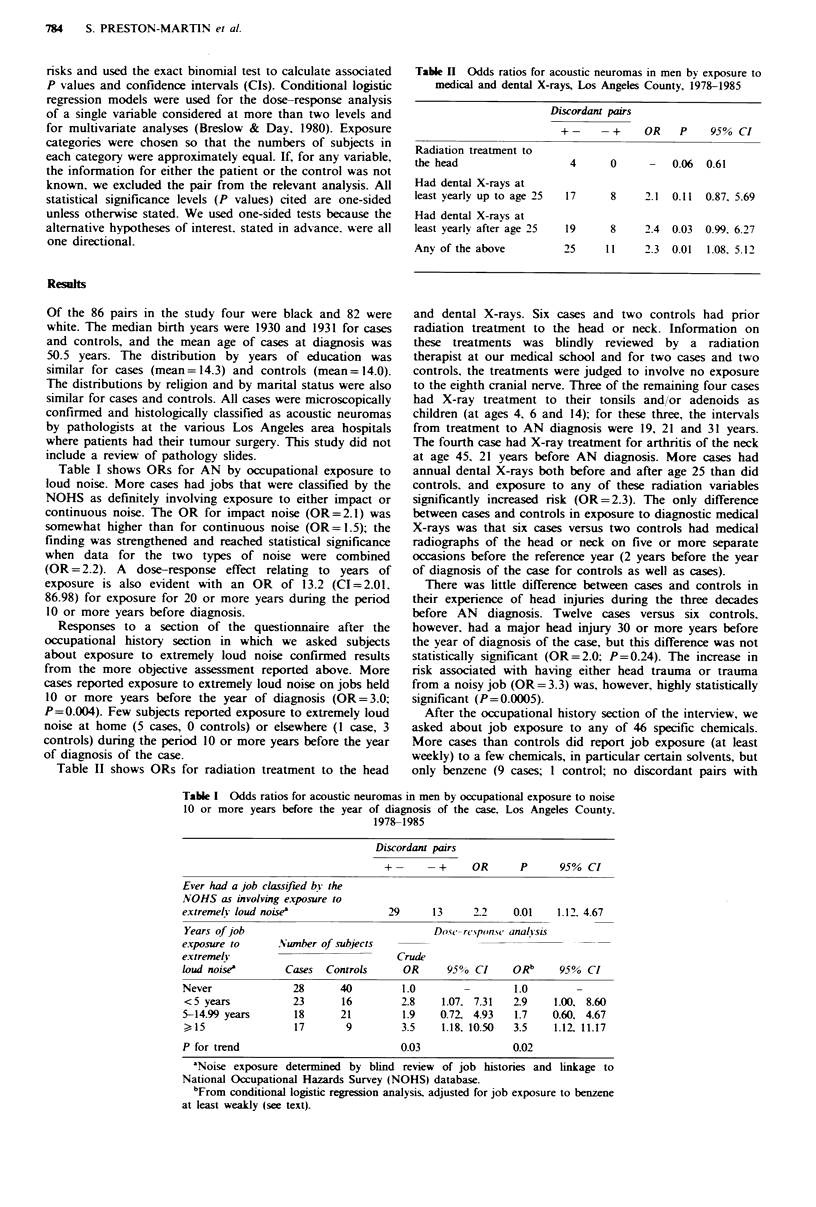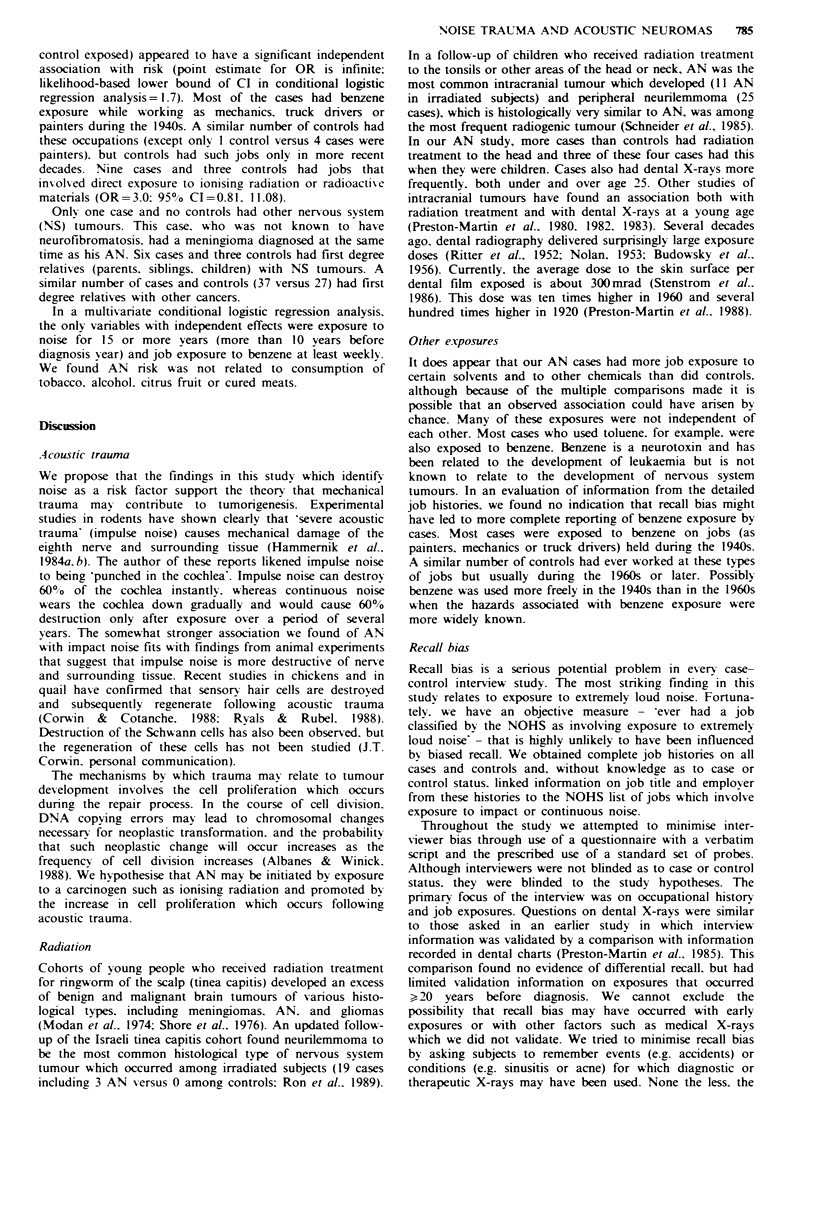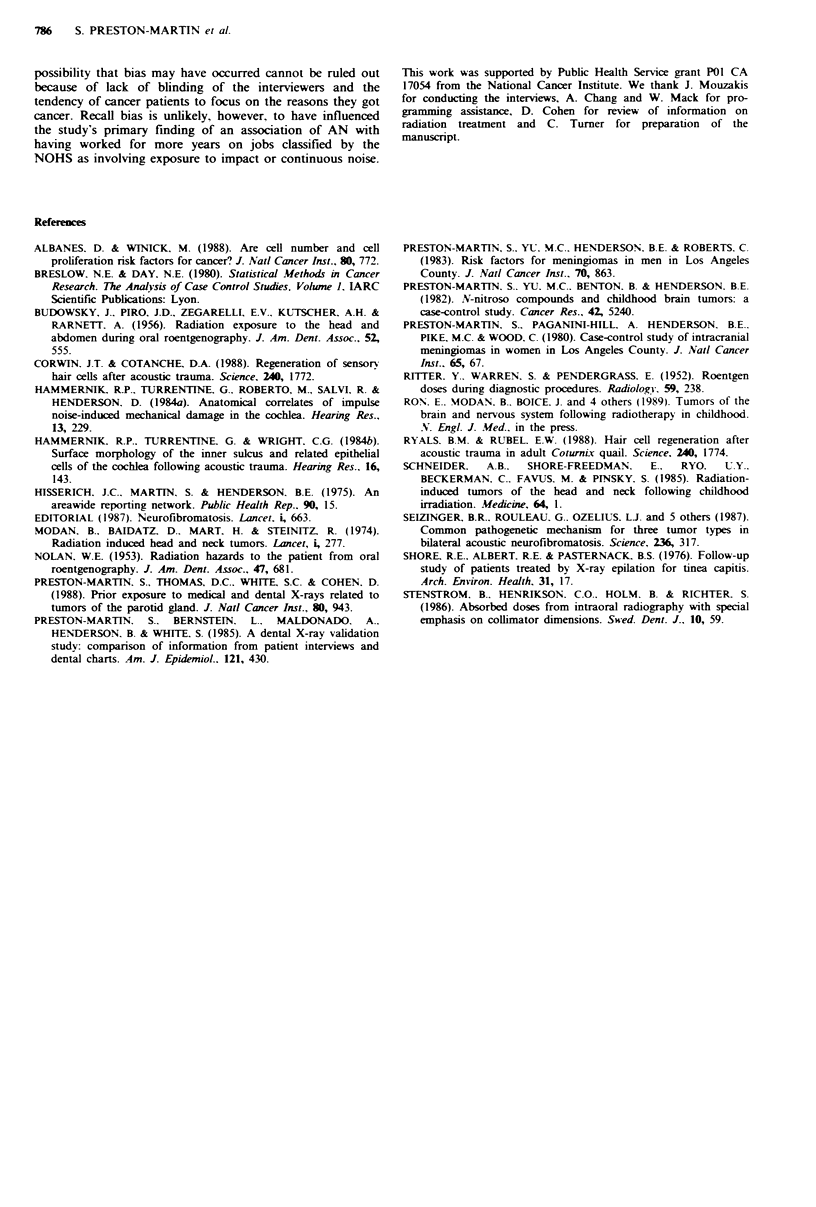# Noise trauma in the aetiology of acoustic neuromas in men in Los Angeles County, 1978-1985.

**DOI:** 10.1038/bjc.1989.163

**Published:** 1989-05

**Authors:** S. Preston-Martin, D. C. Thomas, W. E. Wright, B. E. Henderson

**Affiliations:** Department of Preventive Medicine, University of Southern California School of Medicine, Los Angeles, 90033.

## Abstract

The aim of this study was to investigate whether occupational and other suggested brain tumour risk factors relate to the development of acoustic neuromas (AN) in men. Responses to interviews were compared for 86 AN patients and 86 neighbourhood controls. During the period 10 or more years before the year of diagnosis of the case, more cases than controls had a job involving exposure to extremely loud noise; noise exposure was determined by a blinded review of job histories and linkage to the National Occupational Hazards Survey data base (odds ratio (OR) = 2.2, 95% confidence interval (CI) = 1.12, 4.67). A dose-response analysis showed an increase in risk related to number of years of job exposure to extremely loud noise (P for trend = 0.02) with an OR of 13.2 (CI = 2.01, 86.98) for exposure for 20 or more years during the period up to 10 years before diagnosis. We propose that the findings in this study which identify noise as a risk factor support the hypothesis that mechanical trauma may contribute to tumorigenesis.


Br. J. Cancer (1989), 59, 783-786 © The Macmillan Press Ltd., 1989

### Noise trauma in the aetiology of acoustic neuromas in men in Los Angeles County, 1978-1985

S. Preston-Martin, D.C. Thomas, W.E. Wright & B.E. Henderson

*Department of Preventive Medicine, University of Southern California School of Medicine, 2025 Zonal Avenue, Los Angeles, CA 90033, USA.*

**Summary** The aim of this study was to investigate whether occupational and other suggested brain tumour risk factors relate to the development of acoustic neuromas (AN) in men. Responses to interviews were compared for 86 AN patients and 86 neighbourhood controls. During the period 10 or more years before the year of diagnosis of the case, more cases than controls had a job involving exposure to extremely loud noise: noise exposure was determined by a blinded review of job histories and linkage to the National Occupational Hazards Survey data base (odds ratio (OR) = 2.2, 95% confidence interval (CI) = 1.12, 4.67). A dose-response analysis showed an increase in risk related to number of years of job exposure to extremely loud noise (*P* for trend = 0.02) with an OR of 13.2 (CI = 2.01, 86.98) for exposure for 20 or more years during the period up to 10 years before diagnosis. We propose that the findings in this study which identify noise as a risk factor support the hypothesis that mechanical trauma may contribute to tumorigenesis.

Six per cent of all primary central nervous system (CNS) neoplasms in both men and women are neuromas (also called neurilemmomas or schwannomas); almost all of these arise in the eighth cranial nerve and are called acoustic neuromas (AN). These tumours arise from Schwann cells in the nerve sheath and are slow growing and histologically benign. These tumours can occur in association with either of two genetic syndromes, bilateral acoustic neurofibromatosis (Seizinger *et al.*, 1987) and von Recklinghausen's neurofibromatosis (Editorial, 1987) but only a small minority of AN patients have either of these disorders. None of the patients in our study were known to have bilateral acoustic neuromas or neurofibromatosis. So far as we know, our study is the first to investigate the aetiology of acoustic neuromas which occur in the absence of concomitant inherited disease.

### Subjects and methods

### *Cases*

The patients were black and white men with AN first diagnosed during 1978-1985. Any man who was a resident of Los Angeles County and 25-69 years of age at the time his AN was diagnosed was eligible for inclusion if he was alive and able to be interviewed. The Los Angeles County Cancer Surveillance Program identified the cases (Hisserich *et al.*, 1975). All diagnoses had been microscopically confirmed.

A total of 118 AN patients were identified. The hospital and attending physician granted us permission to contact 106 (90%) patients. We were unable to locate seven patients, and another nine chose not to participate. We interviewed 90 patients (91% of the 99 patients contacted about the study or 76% of the initial 118 patients). There were no otherwise eligible AN patients who were deceased.

### *Controls*

We sought a neighbourhood control for each of these 90 patients by use of a procedure that defines a sequence of houses on specified neighbourhood blocks. Our goal was to interview the first male resident in the sequence who corresponded to the patient in race and age (birth year within 5 years of birth year of the patient). If no one was home at the time of the visit, we left a return envelope, an explanatory letter and a brief questionnaire about the age, sex and race of household members at the residence and made a follow-up visit after several days. In 69 instances, the first appropriate person agreed to be interviewed. In 17 neighbourhoods the first match refused, but we were able to locate and interview another matched control in the sequence. For any patient, we visited 40 housing units and made three return visits before we conceded failure to secure a matched control. We were unable to obtain a control in four neighbourhoods. In all, we identified and interviewed 86 controls. These 86 controls and the corresponding 86 cases were included in the analysis.

### *Questionnaire, interview and coding*

A questionnaire sought information on various life experiences that had occurred 2 years or more before the year of diagnosis of the case. The first and longest section obtained a detailed job history including information on specific job tasks and materials used. A specific list of chemicals and other exposures (radiation, radioactive materials) was also queried in relation to all jobs. Later in the interview, questions were asked about exposure to extremely loud noise at work, at home or elsewhere. There were also questions about head trauma; head X-rays; relatives with nervous system tumours or cancer and consumption of tobacco, alcohol and certain foods. It was not feasible for interviews to be conducted blindly, but all questions were asked in a standard manner. Both members (case and control) of each matched pair were interviewed by the same interviewer and both were interviewed by the same method, either in person or by telephone. Interviews were conducted from August 1979 through December 1986. One of us (W.E.W., a physician in the Division of Occupational Health in our department) blindly reviewed the occupational histories of all cases and controls and for each job determined whether or not significant noise exposure occurred and, if it did, whether it was impact noise or continuous noise. These determinations were made with reference to the National Occupational Hazards Survey (NOHS), noting the job title, employer and job description. The NOHS identified jobs which involve definite or potential exposure to either impact or continuous noise. We coded a job as involving noise exposure only if the NOHS classified it as definitely involving noise.

### *Statistical analysis*

In the analysis of questionnaire data, we used matched odds ratios (OR, the ratio of discordant pairs) to estimate relative

Correspondence: S. Preston-Martin.
Received 25 October 1988, and in revised form, 14 December 1988.

risks and used the exact binomial test to calculate associated $P$ values and confidence intervals (CIs). Conditional logistic regression models were used for the dose–response analysis of a single variable considered at more than two levels and for multivariate analyses (Breslow & Day, 1980). Exposure categories were chosen so that the numbers of subjects in each category were approximately equal. If, for any variable, the information for either the patient or the control was not known, we excluded the pair from the relevant analysis. All statistical significance levels ($P$ values) cited are one-sided unless otherwise stated. We used one-sided tests because the alternative hypotheses of interest, stated in advance, were all one directional.

**Table II** Odds ratios for acoustic neuromas in men by exposure to medical and dental X-rays, Los Angeles County, 1978–1985

| | *Discordant pairs* | | | | |
|---|---|---|---|---|---|
| | + − | − + | *OR* | *P* | *95% CI* |
| Radiation treatment to the head | 4 | 0 | – | 0.06 | 0.61 |
| Had dental X-rays at least yearly up to age 25 | 17 | 8 | 2.1 | 0.11 | 0.87, 5.69 |
| Had dental X-rays at least yearly after age 25 | 19 | 8 | 2.4 | 0.03 | 0.99, 6.27 |
| Any of the above | 25 | 11 | 2.3 | 0.01 | 1.08, 5.12 |

### Results

Of the 86 pairs in the study four were black and 82 were white. The median birth years were 1930 and 1931 for cases and controls, and the mean age of cases at diagnosis was 50.5 years. The distribution by years of education was similar for cases (mean = 14.3) and controls (mean = 14.0). The distributions by religion and by marital status were also similar for cases and controls. All cases were microscopically confirmed and histologically classified as acoustic neuromas by pathologists at the various Los Angeles area hospitals where patients had their tumour surgery. This study did not include a review of pathology slides.

Table I shows ORs for AN by occupational exposure to loud noise. More cases had jobs that were classified by the NOHS as definitely involving exposure to either impact or continuous noise. The OR for impact noise (OR = 2.1) was somewhat higher than for continuous noise (OR = 1.5); the finding was strengthened and reached statistical significance when data for the two types of noise were combined (OR = 2.2). A dose–response effect relating to years of exposure is also evident with an OR of 13.2 (CI = 2.01, 86.98) for exposure for 20 or more years during the period 10 or more years before diagnosis.

Responses to a section of the questionnaire after the occupational history section in which we asked subjects about exposure to extremely loud noise confirmed results from the more objective assessment reported above. More cases reported exposure to extremely loud noise on jobs held 10 or more years before the year of diagnosis (OR = 3.0; $P$ = 0.004). Few subjects reported exposure to extremely loud noise at home (5 cases, 0 controls) or elsewhere (1 case, 3 controls) during the period 10 or more years before the year of diagnosis of the case.

Table II shows ORs for radiation treatment to the head and dental X-rays. Six cases and two controls had prior radiation treatment to the head or neck. Information on these treatments was blindly reviewed by a radiation therapist at our medical school and for two cases and two controls, the treatments were judged to involve no exposure to the eighth cranial nerve. Three of the remaining four cases had X-ray treatment to their tonsils and/or adenoids as children (at ages 4, 6 and 14); for these three, the intervals from treatment to AN diagnosis were 19, 21 and 31 years. The fourth case had X-ray treatment for arthritis of the neck at age 45, 21 years before AN diagnosis. More cases had annual dental X-rays both before and after age 25 than did controls, and exposure to any of these radiation variables significantly increased risk (OR = 2.3). The only difference between cases and controls in exposure to diagnostic medical X-rays was that six cases versus two controls had medical radiographs of the head or neck on five or more separate occasions before the reference year (2 years before the year of diagnosis of the case for controls as well as cases).

There was little difference between cases and controls in their experience of head injuries during the three decades before AN diagnosis. Twelve cases versus six controls, however, had a major head injury 30 or more years before the year of diagnosis of the case, but this difference was not statistically significant (OR = 2.0; $P$ = 0.24). The increase in risk associated with having either head trauma or trauma from a noisy job (OR = 3.3) was, however, highly statistically significant ($P$ = 0.0005).

After the occupational history section of the interview, we asked about job exposure to any of 46 specific chemicals. More cases than controls did report job exposure (at least weekly) to a few chemicals, in particular certain solvents, but only benzene (9 cases; 1 control; no discordant pairs with

**Table I** Odds ratios for acoustic neuromas in men by occupational exposure to noise 10 or more years before the year of diagnosis of the case, Los Angeles County, 1978–1985

| | *Discordant pairs* | | | | |
|---|---|---|---|---|---|
| | + − | − + | *OR* | *P* | *95% CI* |
| *Ever had a job classified by the NOHS as involving exposure to extremely loud noise*[a] | 29 | 13 | 2.2 | 0.01 | 1.12, 4.67 |

| *Years of job exposure to extremely loud noise*[a] | *Number of subjects* | | *Dose–response analysis* | | | |
|---|---|---|---|---|---|---|
| | *Cases* | *Controls* | *Crude OR* | *95% CI* | *OR*[b] | *95% CI* |
| Never | 28 | 40 | 1.0 | – | 1.0 | – |
| <5 years | 23 | 16 | 2.8 | 1.07, 7.31 | 2.9 | 1.00, 8.60 |
| 5–14.99 years | 18 | 21 | 1.9 | 0.72, 4.93 | 1.7 | 0.60, 4.67 |
| ≥15 | 17 | 9 | 3.5 | 1.18, 10.50 | 3.5 | 1.12, 11.17 |
| *P* for trend | | | 0.03 | | 0.02 | |

[a]Noise exposure determined by blind review of job histories and linkage to National Occupational Hazards Survey (NOHS) database.
[b]From conditional logistic regression analysis, adjusted for job exposure to benzene at least weakly (see text).

control exposed) appeared to have a significant independent association with risk (point estimate for OR is infinite; likelihood-based lower bound of CI in conditional logistic regression analysis = 1.7). Most of the cases had benzene exposure while working as mechanics, truck drivers or painters during the 1940s. A similar number of controls had these occupations (except only 1 control versus 4 cases were painters), but controls had such jobs only in more recent decades. Nine cases and three controls had jobs that involved direct exposure to ionising radiation or radioactive materials (OR = 3.0; 95% CI = 0.81, 11.08).

Only one case and no controls had other nervous system (NS) tumours. This case, who was not known to have neurofibromatosis, had a meningioma diagnosed at the same time as his AN. Six cases and three controls had first degree relatives (parents, siblings, children) with NS tumours. A similar number of cases and controls (37 versus 27) had first degree relatives with other cancers.

In a multivariate conditional logistic regression analysis, the only variables with independent effects were exposure to noise for 15 or more years (more than 10 years before diagnosis year) and job exposure to benzene at least weekly. We found AN risk was not related to consumption of tobacco, alcohol, citrus fruit or cured meats.

### Discussion

### *Acoustic trauma*

We propose that the findings in this study which identify noise as a risk factor support the theory that mechanical trauma may contribute to tumorigenesis. Experimental studies in rodents have shown clearly that 'severe acoustic trauma' (impulse noise) causes mechanical damage of the eighth nerve and surrounding tissue (Hammernik *et al.*, 1984*a*, *b*). The author of these reports likened impulse noise to being 'punched in the cochlea'. Impulse noise can destroy 60% of the cochlea instantly, whereas continuous noise wears the cochlea down gradually and would cause 60% destruction only after exposure over a period of several years. The somewhat stronger association we found of AN with impact noise fits with findings from animal experiments that suggest that impulse noise is more destructive of nerve and surrounding tissue. Recent studies in chickens and in quail have confirmed that sensory hair cells are destroyed and subsequently regenerate following acoustic trauma (Corwin & Cotanche, 1988; Ryals & Rubel, 1988). Destruction of the Schwann cells has also been observed, but the regeneration of these cells has not been studied (J.T. Corwin, personal communication).

The mechanisms by which trauma may relate to tumour development involves the cell proliferation which occurs during the repair process. In the course of cell division, DNA copying errors may lead to chromosomal changes necessary for neoplastic transformation, and the probability that such neoplastic change will occur increases as the frequency of cell division increases (Albanes & Winick, 1988). We hypothesise that AN may be initiated by exposure to a carcinogen such as ionising radiation and promoted by the increase in cell proliferation which occurs following acoustic trauma.

### *Radiation*

Cohorts of young people who received radiation treatment for ringworm of the scalp (tinea capitis) developed an excess of benign and malignant brain tumours of various histological types, including meningiomas, AN, and gliomas (Modan *et al.*, 1974; Shore *et al.*, 1976). An updated follow-up of the Israeli tinea capitis cohort found neurilemmoma to be the most common histological type of nervous system tumour which occurred among irradiated subjects (19 cases including 3 AN versus 0 among controls; Ron *et al.*, 1989). In a follow-up of children who received radiation treatment to the tonsils or other areas of the head or neck, AN was the most common intracranial tumour which developed (11 AN in irradiated subjects) and peripheral neurilemmoma (25 cases), which is histologically very similar to AN, was among the most frequent radiogenic tumour (Schneider *et al.*, 1985). In our AN study, more cases than controls had radiation treatment to the head and three of these four cases had this when they were children. Cases also had dental X-rays more frequently, both under and over age 25. Other studies of intracranial tumours have found an association both with radiation treatment and with dental X-rays at a young age (Preston-Martin *et al.*, 1980, 1982, 1983). Several decades ago, dental radiography delivered surprisingly large exposure doses (Ritter *et al.*, 1952; Nolan, 1953; Budowsky *et al.*, 1956). Currently, the average dose to the skin surface per dental film exposed is about 300 mrad (Stenstrom *et al.*, 1986). This dose was ten times higher in 1960 and several hundred times higher in 1920 (Preston-Martin *et al.*, 1988).

### *Other exposures*

It does appear that our AN cases had more job exposure to certain solvents and to other chemicals than did controls, although because of the multiple comparisons made it is possible that an observed association could have arisen by chance. Many of these exposures were not independent of each other. Most cases who used toluene, for example, were also exposed to benzene. Benzene is a neurotoxin and has been related to the development of leukaemia but is not known to relate to the development of nervous system tumours. In an evaluation of information from the detailed job histories, we found no indication that recall bias might have led to more complete reporting of benzene exposure by cases. Most cases were exposed to benzene on jobs (as painters, mechanics or truck drivers) held during the 1940s. A similar number of controls had ever worked at these types of jobs but usually during the 1960s or later. Possibly benzene was used more freely in the 1940s than in the 1960s when the hazards associated with benzene exposure were more widely known.

### *Recall bias*

Recall bias is a serious potential problem in every case–control interview study. The most striking finding in this study relates to exposure to extremely loud noise. Fortunately, we have an objective measure – 'ever had a job classified by the NOHS as involving exposure to extremely loud noise' – that is highly unlikely to have been influenced by biased recall. We obtained complete job histories on all cases and controls and, without knowledge as to case or control status, linked information on job title and employer from these histories to the NOHS list of jobs which involve exposure to impact or continuous noise.

Throughout the study we attempted to minimise interviewer bias through use of a questionnaire with a verbatim script and the prescribed use of a standard set of probes. Although interviewers were not blinded as to case or control status, they were blinded to the study hypotheses. The primary focus of the interview was on occupational history and job exposures. Questions on dental X-rays were similar to those asked in an earlier study in which interview information was validated by a comparison with information recorded in dental charts (Preston-Martin *et al.*, 1985). This comparison found no evidence of differential recall, but had limited validation information on exposures that occurred $\geqslant 20$ years before diagnosis. We cannot exclude the possibility that recall bias may have occurred with early exposures or with other factors such as medical X-rays which we did not validate. We tried to minimise recall bias by asking subjects to remember events (e.g. accidents) or conditions (e.g. sinusitis or acne) for which diagnostic or therapeutic X-rays may have been used. None the less, the possibility that bias may have occurred cannot be ruled out because of lack of blinding of the interviewers and the tendency of cancer patients to focus on the reasons they got cancer. Recall bias is unlikely, however, to have influenced the study's primary finding of an association of AN with having worked for more years on jobs classified by the NOHS as involving exposure to impact or continuous noise.

This work was supported by Public Health Service grant P01 CA 17054 from the National Cancer Institute. We thank J. Mouzakis for conducting the interviews, A. Chang and W. Mack for programming assistance, D. Cohen for review of information on radiation treatment and C. Turner for preparation of the manuscript.

**References**

ALBANES, D. & WINICK, M. (1988). Are cell number and cell proliferation risk factors for cancer? *J. Natl Cancer Inst.*, **80**, 772.

BRESLOW, N.E. & DAY, N.E. (1980). *Statistical Methods in Cancer Research. The Analysis of Case Control Studies, Volume 1*. IARC Scientific Publications: Lyon.

BUDOWSKY, J., PIRO, J.D., ZEGARELLI, E.V., KUTSCHER, A.H. & RARNETT, A. (1956). Radiation exposure to the head and abdomen during oral roentgenography. *J. Am. Dent. Assoc.*, **52**, 555.

CORWIN, J.T. & COTANCHE, D.A. (1988). Regeneration of sensory hair cells after acoustic trauma. *Science*, **240**, 1772.

HAMMERNIK, R.P., TURRENTINE, G., ROBERTO, M., SALVI, R. & HENDERSON, D. (1984*a*). Anatomical correlates of impulse noise-induced mechanical damage in the cochlea. *Hearing Res.*, **13**, 229.

HAMMERNIK, R.P., TURRENTINE, G. & WRIGHT, C.G. (1984*b*). Surface morphology of the inner sulcus and related epithelial cells of the cochlea following acoustic trauma. *Hearing Res.*, **16**, 143.

HISSERICH, J.C., MARTIN, S. & HENDERSON, B.E. (1975). An areawide reporting network. *Public Health Rep.*, **90**, 15.

EDITORIAL (1987). Neurofibromatosis. *Lancet*, **i**, 663.

MODAN, B., BAIDATZ, D., MART, H. & STEINITZ, R. (1974). Radiation induced head and neck tumors. *Lancet*, **i**, 277.

NOLAN, W.E. (1953). Radiation hazards to the patient from oral roentgenography. *J. Am. Dent. Assoc.*, **47**, 681.

PRESTON-MARTIN, S., THOMAS, D.C., WHITE, S.C. & COHEN, D. (1988). Prior exposure to medical and dental X-rays related to tumors of the parotid gland. *J. Natl Cancer Inst.*, **80**, 943.

PRESTON-MARTIN, S., BERNSTEIN, L., MALDONADO, A., HENDERSON, B. & WHITE, S. (1985). A dental X-ray validation study: comparison of information from patient interviews and dental charts. *Am. J. Epidemiol.*, **121**, 430.

PRESTON-MARTIN, S., YU, M.C., HENDERSON, B.E. & ROBERTS, C. (1983). Risk factors for meningiomas in men in Los Angeles County. *J. Natl Cancer Inst.*, **70**, 863.

PRESTON-MARTIN, S., YU, M.C., BENTON, B. & HENDERSON, B.E. (1982). *N*-nitroso compounds and childhood brain tumors: a case-control study. *Cancer Res.*, **42**, 5240.

PRESTON-MARTIN, S., PAGANINI-HILL, A. HENDERSON, B.E., PIKE, M.C. & WOOD, C. (1980). Case-control study of intracranial meningiomas in women in Los Angeles County. *J. Natl Cancer Inst.*, **65**, 67.

RITTER, Y., WARREN, S. & PENDERGRASS, E. (1952). Roentgen doses during diagnostic procedures. *Radiology*, **59**, 238.

RON, E., MODAN, B., BOICE, J. and 4 others (1989). Tumors of the brain and nervous system following radiotherapy in childhood. *N. Engl. J. Med.*, in the press.

RYALS, B.M. & RUBEL, E.W. (1988). Hair cell regeneration after acoustic trauma in adult *Coturnix* quail. *Science*, **240**, 1774.

SCHNEIDER, A.B., SHORE-FREEDMAN, E., RYO, U.Y., BECKERMAN, C., FAVUS, M. & PINSKY, S. (1985). Radiation-induced tumors of the head and neck following childhood irradiation. *Medicine*, **64**, 1.

SEIZINGER, B.R., ROULEAU, G., OZELIUS, L.J. and 5 others (1987). Common pathogenetic mechanism for three tumor types in bilateral acoustic neurofibromatosis. *Science*, **236**, 317.

SHORE, R.E., ALBERT, R.E. & PASTERNACK, B.S. (1976). Follow-up study of patients treated by X-ray epilation for tinea capitis. *Arch. Environ. Health*, **31**, 17.

STENSTROM, B., HENRIKSON, C.O., HOLM, B. & RICHTER, S. (1986). Absorbed doses from intraoral radiography with special emphasis on collimator dimensions. *Swed. Dent. J.*, **10**, 59.